# Comparison of Oral and Intravenous Proton Pump Inhibitor on Patients with High Risk Bleeding Peptic Ulcers: A Prospective, Randomized, Controlled Clinical Trial 

**Published:** 2011-07-01

**Authors:** A A Mostaghni, S A Hashemi, S T Heydari

**Affiliations:** 1 Division of Gastroenterology and Hepatology, Shiraz University of Medical Sciences, Shiraz, Iran; 2 Health Policy Research Center, Shiraz University of Medical Sciences, Shiraz, Iran

**Keywords:** Proton pump inhibitor, Bleeding, Peptic ulce

## Abstract

**Background:**

Proton pump inhibitors (PPIs) decrease the rate of rebleeding following endoscopic hemostatic therapy in patients with bleeding peptic ulcers. This study compares the efficacy of oral omeprazole vs intravenous pantoprazole in decrease of rebleeding of peptic ulcer patients.

**Methods:**

One hundred and six patients with high risk peptic ulcer were randomized to receive either oral omeprazole (80 mg BID for 3 days) or IV pantoprazole (80 mg bolus and 8 mg/hour infusion for 3 days) followed by omeprazole (20 mg each day for 30 days). All patients underwent upper endoscopy and endoscopic therapy within 24 hours.

**Results:**

Seventeen patients were excluded from the study. Forty four patients were randomly allocated into omeprazole group and 41 patients to IV pantoprazole group. Both groups were similar for factors affecting the outcome. Bleeding reoccurred in five patients of omeprazole group and four patients in pantoprazole group (11.4% vs 9.8 %). The mean hospital stay and blood transfusion were not different in both groups.

**Conclusion:**

Oral omeprazole and IV pantoprazole had equal effects on prevention of rebleeding after endoscopic therapy in patients with high risk bleeding peptic ulcers.

## Introduction

Upper gastrointestinal bleeding is a common emergency with significant morbidity and mortality. Peptic ulcer disease is the most common cause accounting for about 50% of episodes.[1][2] Endoscopic therapy of high risk ulcers such as epinephrine injection reduces rebleeding, morbidity and even mortality.[3][4] Therefore, it is currently recommended as the first line of hemostatic intervention for these patients.[4][5] However, high risk ulcers rebleed in 14-36% of patient in spite of efficient endoscopic intervention.[5][6] Gastric acid inhibits clot formation and promotes clot lyses and therefore disturbs hemostasis of ulcers in the stomach and duodenum. So reduction of gastric acid secretion could prevent ulcer rebleeding.[7]

Several controlled trials and meta-analysis studies have shown the efficacy of intravenous and oral proton pump inhibitors (PPIs) in high risk bleeding ulcers after endoscopic therapy.[8][9][10][11][12][13][14][15][16] The comparable effectiveness of oral (PO) and intravenous (IV) route of administration is not well known; therefore a few cost-effectiveness studies were designed, but they show conflicting results and were not conclusive.[17][18][19][20] So to reduce health cost, head to head comparison of these two routes is necessary.

Comparing oral and IV administration of PPI in bleeding peptic ulcers has been studied by Bajaj et al.,[21] and Tsai et al.,[22] however, some problems were encountered in both studies. Bajaj et al. has done a pilot study with small number of patients and Tsai et al. has used regular dose and not high dose of PPI.[21][22] In order to achieve a better evaluation, a prospective, randomized controlled trial was designed to compare oral and intravenous high dose of PPI in high risk peptic ulcer bleeding after endoscopic intervention.

## Materials and Methods

The protocol was approved by the Ethic Committee of Research Department of Shiraz University of Medical Sciences (SUMS) and a written informed consent was obtained from all subjects. From November 2008 to July 2009, all adult patients who were admitted to medical emergency rooms of Faghihi and Nemazee hospitals affiliated to SUMS due to upper gastrointestinal bleeding (as evidenced by hematemesis, melena or hematochezia) were considered for inclusion in the study.

Endoscopy was performed within 24 hours after admission. Patients older than 18 years with successful endoscopic therapy of high risk ulcers [defined as active bleeding (Forrest IA, IB), non- bleeding visible vessel (NBVV, Forrest IIA) or adherent clots (Forrest IIB)] were enrolled. Patients with low risk ulcers (clean base, ulcers with a simple washable clot), suspicious malignant ulcer, bleeding tendency, uremia, liver cirrhosis, Mallory Weiss tear or already on PPI as an outpatient were excluded from study.

The patients with high risk peptic ulcer (Forrest IA-IIB) were managed endoscopically by injecting 5-30 ml of epinephrine (diluted 1:10000) around the ulcer crater to stop bleeding. Cavitation or flattening of bleeding vessel and disappearance of NBVV was considered as established homeostasis. Thereafter, an electrocoagulation therapy by Argon Plasma Coagulation (APC) was applied in some patients with active bleeding and NBVV for further hemostasis.

A biopsy was taken from antrum for evaluating H. pylori infection. Patient with unsuccessful endoscopic therapy were not enrolled and such patients consulted immediately with a general surgeon. A questionnaire (including information on demography, history of previous upper gastrointestinal bleeding, NSAID or ASA ingestion, ulcer location, bleeding stigmata and blood transfusion volume at entry) was completed for all high risk patients.

All the enrolled patients were allocated into two groups to receive either oral omeprazole or IV pantoprazole based on even and odd days of the month. In the omeprazole (OMP) group, the patients received 40 mg omeprazole (Roozdaru Pharmaceutical Co., Tehran, Iran) orally twice daily for 72 hours. In pantoprazole (PAN) group, patients received pantoprazol (NYCOMED Pharmaceutical Co., Germany) 80 mg bolus and then 8 mg/hour infusion for 48-72 hours. Then, all patients received omeprazole, 20 mg orally for 30 days. On the day of discharge H. pylori infected patients were treated using standard regimens.

The patients were monitored for supine and sitting vital signs (BP, PR), intravenous fluid intake, blood transfusion and urine output. Hemoglobin (Hb) was checked every each 8 hours and blood transfusion was done if Hb was lower than 8 g/dl or the patient was in the state of shock. Rebleeding was suspected if persistent tarry stool, reappearance of hematemesis, orthostatic hypotension, unstable vital sign (BP≤90, PR≥120) or Hb drop≥2 g.dl, (despite blood transfusion) developed after the first endoscopic therapy. Patients suspected to rebleeding were evaluated by urgent endoscopy and if active bleeding, fresh blood or blood clots were seen, rebleeding was documented. In such cases, endoscopic therapy with epinephrine injection and electrocoagulation (by Argon plasma coagulation, APC) was done to stop bleeding.

Statistical analysis was performed using statistical analysis of SPSS software (Version 11.5, Chicago, Il, USA). The descriptive variables such as mean, standard deviations and frequency were used. Chi Square (X2) was performed for finding out the association between rebleeding, blood transfusion, reendoscopy and OPM or PAN groups. T-test was performed to difference between hospital stay, amount of blood transfusion and OPM or PAN groups. P value less than 0.05 was considered significant.

## Results

From 209 patients who were referred to Emergency Room due to upper GI bleeding, 102 patients had high risk peptic ulcer in endoscopic evaluation. Seventeen patients were excluded from the study (bleeding tendency=4, uremia=2, gastric cancer=3, Mallory Weiss tear=1, esophageal varices=4, primary endoscopic failure=3). Finally, 85 patients completed the study (44 patients in OMP group and 41 patients in PAN group). Both groups were similar in source of bleeding and factors affecting the outcomes (Table 1).

**Table 1:** Demographic and presentation characteristics of 89 studied patients

	**OMP group (no.= 44)**	**PAN group (no.=41)**
Gender (male/Female)	33/11	30/11
Age (years) (mean ±SD)	57.25±16.45	61.66±17.17
NSAID, ASA use (%)	19 (43)	17 (41)
Ulcer Location		
Gastric (%)	24 (54)	18 (44)
Duodenal (%)	17 (39 )	20 (49)
Both (%)	3 (7 )	3 (7)
Ulcer stigmata		
Adherent clot (%)	5 (11)	3 (7)
Visible Vessel (%)	25 (57)	26 (63)
Blood oozing (%)	8 (18)	5 (13)
Active Bleeding (%)	6 (14)	7 (17)
Therapeutic intervention		
Epinephrine injection alone (%)	29 (66)	28 (68)
Epinephrine + APC (%)	14 (32)	12 (29.5)
Epinephrine + endoclips (%)	1 (2)	1 (2.5)

Five patients in OMP group and 4 in the PAN group rebled (11.4% vs 9.8%) which was not statistically significant (p=0.810) (Table 2). From the 5 rebleeding cases in OMP group, 3 patients rebled in hospital course and were successfully managed by endoscopic epinephrine injection and electrocoagulation (APC). Then, bleeding did not reoccur up to 2 wk follow up. In 2 patients, bleeding developed in 2 weeks after discharge during the follow up. They were managed again with epinephrine injection and APC and bleeding did not reoccur up to 2 weeks after the follow up.

**Table 2:** Primary and secondary end points^a^

	**OMP group (no.= 44)**	**PAN group (no.=41)**	**P value**
Rebleeding (%)	5 (11.4)	4 (9.8)	0.810
Surgery (%)	0 (0)	0 (0)	N.S.
Death (%)	1 (2)	1 (2)	N.S.
Hospital stay (Day)	3.1	3.6	0.130
Blood transfusion (%)	31 (71)	33 (81)	0.284
Amount of blood Transfusion (bag)	1.82	1.95	0.641
Reendoscopy (%)	18 (41)	24(59)	0.104

^a^ N.S: statistically not significant

In PAN group, 4 patients rebled, two of them occurred in hospital course and stopped with epinephrine injection plus APC and recovered uneventfully. The two other patients rebled in follow up period and bleeding stopped with sclerotherapy and APC. Bleeding did not reoccur up to 2 weeks but in one patient 3 weeks later, bleeding reoccurred (5 weeks after index bleeding). So, surgical intervention was done because bleeding continued and endoscopic therapy failed.

One patient in each group died which was due to a comorbid disease and not bleeding. The number of blood transfusion and hospital stay were not statistically different in both groups (Table 2). In none of cases, surgical intervention was done for control of bleeding after endoscopic therapy (except for one patient whose surgery was done 5 weeks after the index bleeding and 3 patients who were operated due to failure of primary endoscopic hemostatic therapy).

The cost of oral and intravenous administration of drugs was calculated, being significantly lower in the OMP group than PAN group (Table 3). Moreover, the mean of the hospital stay in both groups was not statistically different (Table 2) and oral administration of omeprazole led to less hospital stay and costs.

**Table 3:** The cost of oral oral omeprazole and intravenous pantoprazole administration in each patient

	**PAN group**	**OMP group**
Nursing care (Rials)	22,850	2,300
Equipments (Rials)	17,800	-
Drug:		
Stat dose^a^(Rials)	300,000	-
Maintenance for 24 h (Rials)	820,000 b	3,000 c
Total (Rials)	1,160,650	5,300

^a^ Stat dose 80 mg pantoprazole,

^b^ Eight mg each hour,

^c^ Forty mg each 12 hours

## Discussion

Endoscopic therapy decreases but does not eliminates the risk of adverse outcome in peptic ulcer bleeding.[5][6] On the other hand, gastric acid antagonizes hemostasis in the stomach and duodenum by impairing clot formation and promoting clot lysis.[7] So, maintenance of intragastric pH>6 has been considered to result in a lower rebleeding rate of peptic ulcer. In recent years, several studies have shown the efficacy of IV proton pump inhibitors (PPIs) in reducing the adverse outcome of peptic ulcer bleeding, despite the optimal dose, and the best route of administration has remained controversial.[11][12][23] However, IV administration of PPIs has limitations. They are expensive, require a dedicated IV line, need nursing supervision and hospital admission. So, it would be reasonable to prescribe oral PPIs to patients with high risk bleeding ulcers provided that it is as effective as its IV counterpart.

Oral PPIs have a high bioavailability. Its effect initiates one hour after ingestion and the maximal plasma concentration is achieved after 2-3 hours.[24] Several studies have shown similar effectiveness of oral and IV PPIs on rising intragastric pH. Laine et al. in their study comparing frequent oral and IV lanzopiazole have shown that intragastric pH differs only at the first hour of administration and at >1.5 hour, there is no difference among all hourly intragastric pH between both groups.[25] More recently, Javid et al. have demonstrated that IV and high PO doses of various PPIs are equal in their ability to suppress gastric acid secretion and there is no significant difference among various PPIs given through different routes on rising gastric pH above 6 for 72 hours after successful endoscopic hemostasis.[26]

In the clinical setting, many studies have shown that oral PPIs are effective in decreasing the adverse outcomes of high risk bleeding ulcers especially rebleedings.[13][14][15][16] It seems that oral PPI is not less effective than IV PPI. Bardou et al. in their meta-analysis study have concluded that high dose oral PPI following endoscopic treatment significantly decreases rebleeding (-15.3%; 95% CI: -16.5 to -14.0) and probably mortality as compared with placebo.[12]

Andriulli et al. in summarization of several randomized trials have concluded that PPI decreases the adverse outcome of ulcer bleeding independent of the route and dose of PPI.[27] Moreover Leontiadis et al. recently showed that administering oral PPI both before and after endoscopic hemostatic therapy for patients with high risk ulcer bleeding is likely to be the most cost-effective strategy.[19]

However there are few studies comparing oral and IV PPI head to head in clinical setting so for. To the best of our knowledge, only recently Tsai et al. in a randomized control head to head trials comparing oral rabeprazole and IV omeprazole, concluded that both forms of PPI prevented equally rebleeding in patients with high risk peptic ulcers.[22]However in Tsai et al.´s ¬study a high dose of oral PPI has been compared with regular dose of IV PPI (40 mg IV infusion each 12 hours) and not high dose of PPI which is believed (despite controversial results) to be more effective. Therefore there was an attempt in this study to compare oral and IV PPI directly.

Our study revealed that high dose oral omeprazole (40 mg bid) is equally effective as high dose IV pantoprazole (8 mg each hour) in recurrent bleeding, blood transfusion hospital study and mortality after successful endoscopic hemostatic therapy. In comparison with Tsai et al.'s study we achieved a lower rate of rebleeding (overall 10.6% vs. 16%) similar to result obtained by Colvet et al.[28] which could be due to combination endoscopic therapy done as a primary hemostatic procedure. (In 34% of patient combination therapy applied). However our rebleeding rate was higher than that in Lau et al.'s study (10.6 % vs. 6.7%).

Our patients had a high rate of ASA and NSAID consumption (overall 42%) and this may result in higher bleeding rate. But it could be concluded that oral PPIs are also effective in patients who are on ASA or NSAIDS and develop ulcer bleeding. Also, our study revealed that oral administration of PPI drugs are more economical and cost effective than IV administration route. Furthermore, using oral route will decrease personal, pharmaceutical and medical care costs.

Several limitations could be considered in our study. First we administered the drugs on admission and before the endoscopic therapy. Considering the fact that the presence of blood in stomach causes proton pumps activation and their subsequent irreversible deactivation by PPIs, we administered PPI (both oral and IV) on admission and before endoscopic intervention. Currently available evidences have shown that preendoscopic administration of PPIs in patients with non-variceal upper gastrointestinal bleeding downstages the severity of the endoscopic signs of recent bleeding and may reduce the requirement for endoscopic hemostatic therapy at index endoscopy although it does not affect the mortality, rebleeding or surgical intervention rates.[19][23] However our results showed that preendoscopic administration of PPIs affect the adverse outcome in patients with peptic ulcer bleeding.

Second we did not calculate the Rockall score to determine if both groups have equal risk of rebleeding. Still, both groups were matched for factors affecting the adverse outcome. Third we imposed strict exclusion criteria on various factors, so a large number of patients dropped, resulting in the fact that the merit of our study was low for detecting small differences. However surgical intervention and mortality rate is very low in peptic ulcer bleeding due to advanced therapeutic methods applied in recent years and for evaluation of such subtle differences a very large number of patients should be evaluated.

In conclusion, our study revealed that oral high dose PPI is as effective as IV high dose PPI in reducing rebleeding rate, mortality, hospital stay, and blood transfusion after endoscopic therapy of patients who bleed from peptic ulcers and it will be possible to be replaced with IV PPI although further similar studies are recommended to support the result of this study.
